# Statistical Characterization of Wireless Power Transfer via Unmodulated Emission

**DOI:** 10.3390/s22207828

**Published:** 2022-10-14

**Authors:** Sebastià Galmés

**Affiliations:** 1 Departament de Ciències Matemàtiques i Informàtica, Universitat de les Illes Balears, 07122 Palma, Spain; sebastia.galmes@uib.es; 2 Institut d’Investigació Sanitària Illes Balears, 07120 Palma, Spain

**Keywords:** radio-frequency energy harvesting, wireless power transfer, path loss, shadowing, multi-path fading, unmodulated carrier, additive white Gaussian noise, mean, variance, correlation

## Abstract

In the past few years, the ability to transfer power wirelessly has experienced growing interest from the research community. Because the wireless channel is subject to a large number of random phenomena, a crucial aspect is the statistical characterization of the energy that can be harvested by a given device. For this characterization to be reliable, a powerful model of the propagation channel is necessary. The recently proposed generalized-K model has proven to be very useful, as it encompasses the effects of path loss, shadowing, and fast fading for a broad set of wireless scenarios, and because it is analytically tractable. Accordingly, the purpose of this paper is to characterize, from a statistical point of view, the energy harvested by a static device from an unmodulated carrier signal generated by a dedicated source, assuming that the wireless channel obeys the generalized-K propagation model. Specifically, by using simulation-validated analytical methods, this paper provides exact closed-form expressions for the average and variance of the energy harvested over an arbitrary time period. The derived formulation can be used to determine a power transfer plan that allows multiple or even massive numbers of low-power devices to operate continuously, as expected from future network scenarios such as the Internet of things or 5G/6G.

## 1. Introduction

Recent developments in low-power integrated circuits and wireless technologies, the emergence of new application paradigms in the context of the Internet of things (IoT), and a better understanding of propagation phenomena, have led the scientific community to revise Tesla’s initial idea of the wireless power transfer. This idea is now seen as a promising and achievable solution to overcome the limitations of conventional power supply methods, such as batteries or wired connections to fixed power grids. Given the large number of nodes expected to be interconnected in IoT applications and other 5G/6G scenarios, the benefits of radio frequency-based energy harvesting (RF-EH) in terms of operating cost savings and self-sustainability are undoubted. In addition, whether based solely on RF-EH or combined with other primary energy sources (solar radiation, mechanical vibration, air flow, etc.), the panacea of perpetual operation of wireless networks seems somewhat closer today.

Research in RF-EH has already produced significant results, as indicated in recent survey papers. Examples are [1,2,3,4,5,6]. Moreover, relatively new books like [7] or [8] contain good compilations of the main contributions and results. Until now, however, less attention has been paid to the role of the propagation model, either because current work has focused on design aspects or applications of the energy-harvesting technology, or because it has relied on assumptions that neglect the importance of the wireless environment. For example, many papers assume the presence of a dedicated energy source, which performs channel estimation to adjust the transmitted power accordingly. Therefore, the specific propagation model plays a secondary role in these works. However, an important application area for RF-EH is IoT, which is expected to comprise thousands or even millions of extremely simple and low-cost wireless devices. Thus, it is quite possible that these devices will not be able to participate in channel state information (CSI) estimation procedures, nor will the power transmitter be able to cope with the excessive workload involved in keeping track of each connection.

Given the erratic behavior of the wireless channel, the main motivation of this paper is to analyze in detail the impact of the variability of the received power on the amount of electromagnetic energy that can be captured by a device. To fully capture this variability, a key aspect is the selection of a powerful channel propagation model that encompasses all sources of signal variation in a flexible manner. Thus, the main contributions regarding the use of channel propagation models in the characterization of the energy-harvesting process are reviewed below.

First, the propagation models used in RF-EH are outlined in some of the above survey papers. This is the case of [1,6], which includes a brief description of wireless propagation through manageable models such as free-space, two-ray, or Rayleigh fading [4], which recalls the free-space and two-ray models and [5], which only makes reference to the Friis path-loss formula. As for regular papers, most also adopt simplified propagation models. For example, the work presented in [9] assumes free-space path loss and additive white Gaussian noise, which is consistent with the use of a power transmitter mounted on an unmanned autonomous vehicle (UAV) flying along a circular path over the wireless nodes. The objective is to optimize the trajectory radius of the UAV so that a fair allocation of energy between the participating devices is achieved. In [10], a two-ray model is used to account for both line of sight (LOS) and non-line of sight (NLOS) propagation. The analysis focuses on the impact of the NLOS component and other factors (radiation pattern of the transmit and receive antennas, losses associated with different polarization of transmitting field, and efficiency of the power harvester circuit) on the average energy harvested. The use of the two-ray model applies to situations in which there is a direct LOS component and a clear NLOS component reflected by a uniform ground plane. This model is further extended to include path loss, lognormal shadowing, and Rician fading. The latter characterizes the aggregation of many weak scattered rays rather than a single dominant one. The paper then focuses on the estimation of the path loss and shadowing parameters as well as the Rician factor on the basis of experimental measurements. The paper concludes with the assertion that the Rayleigh fading model is not well suited to characterize practical scenarios of RF-EH due to the presence of a strong LOS component. Reference [11] discusses a power beamforming strategy for distributed power transfer from multiple transmitters to a single receiver, based on a relatively simple path-loss model combined with Rayleigh fading and Gaussian noise. Another example is [12], which characterizes the channel by a Nakagami-m fading model. Its purpose is to determine an optimal transmission policy for the RF energy-harvesting device, which switches between two modes: on and off. In the on mode, the device is operational and powered by a battery; in the off mode, the device turns off, and the battery feeding process stops. In [13], various empirical path-loss models are used to determine the usability and fundamental limits of joint RF and photovoltaic harvesting-based M2M communications. Essentially, the theoretical bounds derived in this paper are based on the well-known Shannon’s capacity theorem. In [14], several empirical path-loss models are used with the objective of proposing design guidelines for all stages of the end-to-end RF-to-DC energy-conversion process. In [15], both the Nakagami-m and generalized-K fading models are considered in the statistical characterization of the battery recharging time. In [16], the HATA model, the Ericsson model, and the ITU-R model are used to provide outdoor RF spectral survey results in suburban areas. Basically, the main objective of this paper is to determine the frequency bands most suitable for energy harvesting from RF ambient sources in populated environments.

Among the reviewed literature, the closest contributions to the work presented in this paper are made by [17,18]. The first characterizes the average and variance of the energy collected by a node from the RF signal generated by multiple transmitters distributed according to a spatial Poisson process. The channel between each transmitter and the collector node is initially assumed to be generalized-K, but it is then approximated by a gamma distribution and finally mixed with the rest of the channel distributions to produce a general Gaussian process according to the central limit theorem. Very general propagation models are also considered in [18], such as the generalized η-μ and κ-μ models, which are used to obtain exact closed-form expressions for the distribution, mean, variance, and higher-order moments of the recharge time. However, no relationship is provided between the mathematical parameters of these models and the physical parameters of the system.

This paper fills the previous gaps by fully considering the generalized-K propagation model for the statistical characterization of the energy collected by a single device over an arbitrary time interval. The generalized K-model is doubly advantageous in that it is analytically tractable (in contrast to what is stated in [17]) while covering a wide variety of scenarios with respect to all perturbations introduced by wireless channels, namely path loss, shadowing, and fast fading. Specifically, the main contributions of this paper can be listed as follows.

- Exact closed-form expressions are obtained for the expectation and variance of the energy harvested by a static device, which is assumed to be illuminated by a dedicated power source emitting an unmodulated carrier (a WPT system is thus considered). To the author’s knowledge, this is the first work that adopts such a very general propagation model to accurately characterize the statistics of the RF energy-harvesting process.
- A detailed evaluation is performed showing the sensitivity of the statistical parameters with respect to the physical parameters of the propagation environment (transmission distance, path-loss exponent, shadowing spread, and Nakagami parameter).

Table 1 summarizes the current state-of-the art work in RF-EH related to the specification and use of channel propagation models.

In the context of RF energy harvesting, one of the paradigms with the greatest projection is the wireless energy network (WEN) [19]. The results obtained in this paper are useful in several aspects related to the planning and performance of this type of network.

- To define areas with different levels of energy coverage, once a potential location of the primary and secondary power sources is defined. Here, a grade of service metric such as the probability of energy outage will be of the utmost importance.
- To analyze the queuing time experienced by the energy requests directed to the same power source.

The rest of the paper is organized as follows. In Section 2, the basics of RF-EH are reviewed. In Section 3, the problem is formulated by stating the system model and assumptions, the energy harvesting equations and the generalized-K distribution. Exact closed-form expressions for the average and variance of the energy harvested by a static device are respectively obtained in Section 4 and Section 5. In Section 6, the analytical expressions are validated by simulation and then numerical results are obtained. Finally, in Section 7, the main conclusions and suggestions for further research are drawn.

## 2. Fundamentals of RF-EH

RF-EH has recently emerged as a disruptive technology that allows low-power portable devices and energy-constrained wireless networks to convert the electromagnetic energy present in the environment into DC current. This idea is not new, as it dates back to the early years of the last century, when Nikola Tesla designed and built an experimental station (Wardenclyffe Tower) for the wireless transfer of information and energy to remote devices. However, the project did not receive enough funds and was quickly abandoned before it became operational due to several reasons: the low efficiency of the electric-to-electromagnetic-to-electric conversion process as well as health concerns related to high-power transmitters. Fortunately, in the past few years we have witnessed a resurgence of the concept, as a result of its reformulation for low-power wireless devices.

Wireless power transfer (WPT) techniques fall into one of two major categories, namely near field and far field. The distinction is made because electromagnetic waves behave very differently in these two regions, and correspondingly the techniques to collect energy from them are also quite different. Near-field propagation takes place within an area of about one wavelength of the transmitting antenna, which typically corresponds to distances of at most several meters. As detailed in [3], propagation in the near-field region is essentially non-radiative, meaning that power leaves the transmitter only when there is a receiver to couple to within such a region. Accordingly, power can be transferred in the near-field region by employing inductive coupling, capacitive coupling or their enhanced versions, which consist of adding resonant circuits in order to increase the power-coupling coefficient and, consequently, the transmission range. However, for distances of hundreds of meters or even several kilometers, far field is the only possible region of operation. In contrast to near-field propagation, far-field propagation is radiative, and it obeys the well-known Friis equation when no obstacles are present between transmitter and receiver. RF-EH encompasses systems and techniques devoted to far-field WPT via electromagnetic signals like radio waves, microwaves, or light waves.

**Figure 1:**
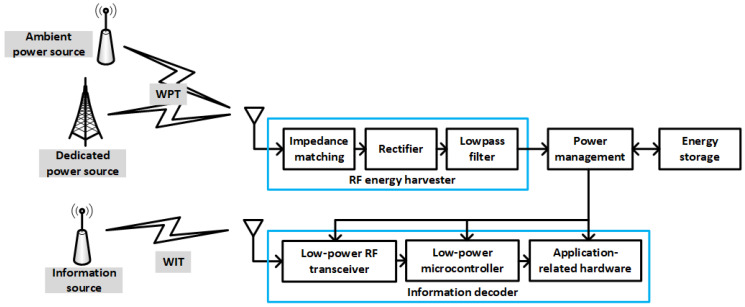
WIPT scenario, with separated WIT and WPT segments. The WIT segment contains conventional hardware to receive information, whereas the WPT segment is essentially a voltage rectifier followed by a low-pass filter devoted to extracting the DC component of the received signal.

Various architectures have been proposed for energy-harvesting systems that coexist with traditional data receivers. One solution is to have independent segments for WPT and wireless information transfer (WIT), as depicted in Figure 1. Such a global scenario of wireless information and power transfer (WIPT) is also referred to as separated receiver architecture. As can also be seen, power sources can be classified into two classes: dedicated power sources and ambient power sources. Dedicated power sources are specifically deployed to transfer RF energy to one or several nodes. These sources can use the license-free ISM frequency bands, though subject to restrictive upper bounds on transmission power. On the other hand, ambient power sources, that is, transmitters that are not intended for RF energy transfer, are always available at no cost, but the collected energy can be very small. Consequently, for applications that require stable and predictable energy supply, dedicated power sources are preferable.

**Figure 2:**
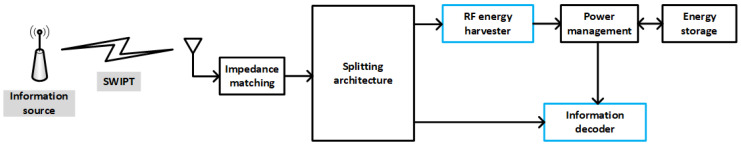
SWIPT scenario. Power is obtained from the same information signal, by using a splitting architecture that generates an input for every independent process.

The architecture represented in Figure 1 is also known as out-of-band RF energy harvesting, because the node collects energy from an RF signal different from that used to receive information. On the other hand, because the information signal also carries energy, a new modality called simultaneous wireless information and power transfer (SWIPT), or in-band energy harvesting, was devised. The new architecture is represented in Figure 2. As can be seen, SWIPT allows for use of a single antenna (or antenna array) to obtain both information and energy. However, a splitting architecture is required in order to distribute the received signal among the two processes. The reason is that a serial implementation would not be feasible regardless of which process was implemented first. Either the energy harvester would destroy information, or the information decoder would consume all signal power. References [1,3] provide very complete descriptions of the WIPT and SWIPT architectures.

**Figure 3:**
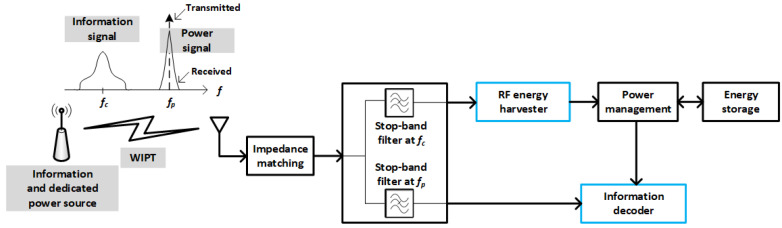
Frequency-splitting architecture. This architecture is really a variant of WIPT, because information and power are transferred via separated signals in the frequency domain, but just a single antenna is required at each side of the link (as in SWIPT).

Finally, another architecture is frequency splitting, the diagram of which is shown in Figure 3. In this architecture, the input signal transports both information and power by using separated frequencies. In fact, this signal consists of the information-bearing modulated component plus an unmodulated sine wave that essentially results from shifting the carrier frequency used in the modulation process, from a value $f_{c}$ to a different value $f_{p}$. The figure also highlights the fact that though the transmitter sends a pure sinusoidal to transfer power, the received signal exhibits some spread due to variations in the channel coefficient. Consequently, there must be a sufficiently high guard band between $f_{c}$ and $f_{p}$.

## 3. Problem Formulation

In this section, the system model and assumptions, the fundamental energy harvesting equation and the generalized-K distribution are formulated.

### 3.1. System Model and Assumptions

**Figure 4:**
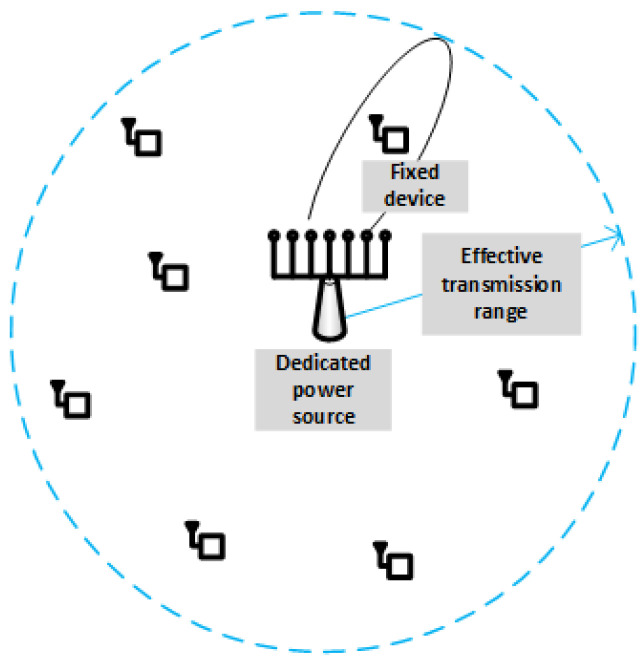
System model.

As stated in [1], the SWIPT architectures always impose a trade-off between information rate and amount of RF energy harvested. To bypass this trade-off, especially in situations where a stable and predictable energy supply is required, WPT from a dedicated source is preferable. Accordingly, this paper focuses on scenarios like those shown in Figure 1 or Figure 3. More specifically, the system under analysis is shown in Figure 4, where a dedicated power source generates an unmodulated carrier at frequency $f_{p}$ in the RF, microwave, or visible light bands. Both the power source and the receivers are located at fixed positions and equipped with directional antennas of gains $G_{t}$ and $G_{r}$, respectively. In particular, the power source uses a phased array that allows for tuning the main beam in the direction of current receiver, whereas receiver antennas are always aligned with the power source. An additive white Gaussian noise (AWGN) channel is assumed, with spectral noise density $N_{0}$ and a channel coefficient h(t) that obeys the abovementioned generalized-K distribution. Moreover, the energy-harvesting device is assumed to operate linearly, in spite of the presence of non-linear components like diodes. For the sake of generality, the analysis will start from an arbitrary waveform x(t) for the power-bearing signal, and only in the end will it be particularized to the special case of an unmodulated carrier. The corresponding power transfer bandwidth is *B*, meaning that the impedance matching circuit (or the combined effect of this circuit and the stop-band filter at $f_{c}$ if the scenario of Figure 3 is considered) has an equivalent bandwidth *B* centered at frequency $f_{p}$.

### 3.2. Energy-Harvesting Equation

Using complex notation, the power-bearing signal x(t) can be expressed in terms of its equivalent low-pass signal or complex envelope $\tilde{x}\left(t\right)$ as follows:

(1) $$x\left(t\right)=ℜ\left\{\tilde{x}\left(t\right)\cdot e^{j2\pi f_{p}t}\right\}.$$

Here, *ℜ* stands for the real part. As pointed out in [20], the received signal can be generally formulated in this way:

(2) $$r\left(t\right)=ℜ\left\{\sum_{k=0}^{K(t)}\alpha_{k}\left(t\right)\cdot \tilde{x}\left(t-\tau_{k}\left(t\right)\right)\cdot e^{j\left(2\pi f_{p}\left(t-\tau_{k}\left(t\right)\right)+\varphi_{k}\right)}\right\}+n\left(t\right).$$

In this expression, n(t) is the AWGN component, K(t) is the total number of resolvable multi-path components and $\alpha_{k}\left(t\right)$, $\tau_{k}\left(t\right)$, and $\phi_{k}$ are, respectively, the time-dependent amplitude and delay parameters and a constant phase offset associated with the *k*:th resolvable multi-path component. If the delay spread of the channel, $T_{m}$, verifies $T_{m}\ll \frac{1}{B}$, then $\tilde{x}\left(t-\tau_{k}\left(t\right)\right)≅\tilde{x}\left(t\right)$. This is the so-called narrow-band fading condition. Note that for x(t) consisting of an unmodulated carrier, this condition holds for any $T_{m}$. Consequently, under narrow-band fading, we have [20]

(3) $$r\left(t\right)=ℜ\left\{\left(\sum_{k=0}^{K(t)}\alpha_{k}\left(t\right)\cdot e^{-j\left(2\pi f_{p}\tau_{k}\left(t\right)-\varphi_{k}\right)}\right)\cdot \tilde{x}\left(t\right)\cdot e^{j2\pi f_{p}t}\right\}+n\left(t\right).$$

The summation term between parentheses is independent of the complex envelope $\tilde{x}\left(t\right)$, and hence it simply introduces a multiplicative effect on the signal. It is the so-called channel coefficient, typically denoted by h(t). Accordingly, Equation (3) can be rewritten in the following way:

(4) $$r\left(t\right)=ℜ\left\{\tilde{x}\left(t\right)\cdot h\left(t\right)\cdot e^{j2\pi f_{p}t}\right\}+n\left(t\right).$$

The noise component can also be reformulated in terms of its complex envelope $\tilde{n}\left(t\right)$ as $n\left(t\right)=ℜ\{\tilde{n}\left(t\right)\cdot e^{j2\pi f_{p}t}\}$, and therefore the channel output signal can be expressed as follows:

(5) $$r\left(t\right)=ℜ\left\{\left(\tilde{x}\left(t\right)\cdot h\left(t\right)+\tilde{n}\left(t\right)\right)\cdot e^{j2\pi f_{p}t}\right\}.$$

The term multiplying the exponential is nothing more than the complex envelope of the received signal r(t), namely $\tilde{r}\left(t\right)$:

(6) $$\tilde{r}\left(t\right)=\tilde{x}\left(t\right)\cdot h\left(t\right)+\tilde{n}\left(t\right).$$

**Figure 5:**
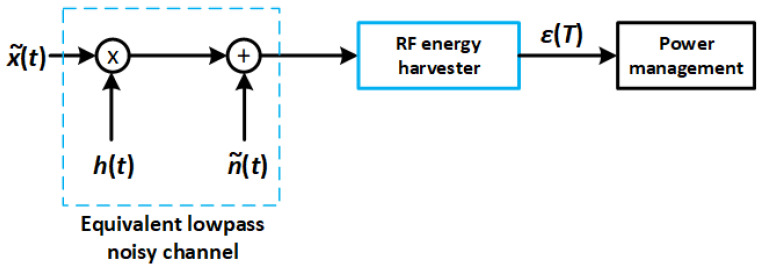
Equivalent low-pass model of the channel.

Figure 5 shows the equivalent low-pass representation of the channel effect, as well as the subsequent energy-harvesting and power-management units. The energy-harvesting unit is capable of producing a certain amount of energy at the end of an exposition time period *T*. This energy, denoted as E(T), can be mathematically formulated as follows:

(7) $$E\left(T\right)=\eta \int_{T}r^{2}\left(t\right)\cdot dt.$$

Here, the integral represents the RF energy captured by the receiving antenna along the period *T*, whereas η denotes the RF-to-DC conversion efficiency. Equation (7) constitutes the starting point of the analysis performed in this paper.

### 3.3. The Generalized-K Distribution

The generalized-K distribution is a powerful description of the perturbation effects experienced by signals in wireless propagation environments. It was proposed in the early 2000s as a compound probability density function (PDF) encompassing all sources of wireless signal degradation, namely path loss, shadowing, and fast fading. In addition, it enjoys the property of analytical tractability, which helps to obtain closed-form expressions for the performance measures of interest. An example is the statistical characterization of the energy harvesting performed in this paper. The availability of closed-form expressions for magnitudes such as the mean and variance of the energy harvested is very useful for network planning, especially in scenarios like IoT, where the energy provider cannot rely on CSI to adjust its transmission power because of the expected massive number of participating devices.

The generalized-K model combines the Nakagami-*m* distribution for fast fading and the gamma distribution for path loss and shadowing (Nakagami gamma). In addition, it can be particularized to numerous well-known models, such as Rayleigh lognormal (Suzuki model), Nakagami lognormal, Rayleigh gamma (K model) and, in an approximate way, Rician gamma, and Rician lognormal.

Let z=z(t)=∥h(t)∥ be the envelope of the channel coefficient. Assuming that the channel is stationary, *z* can be treated as a single random variable whose statistical characterization is independent of time. If this characterization obeys the generalized-K model, the probability density function (PDF) of *z* can be expressed as follows [21]:

(8) $$f_{Z}\left(z\right)=\frac{4\sqrt{\frac{m}{b}}}{\Gamma (a)\Gamma (m)}{\left(\sqrt{\frac{m}{b}}z\right)}^{a+m-1}K_{a-m}\left(2\sqrt{\frac{m}{b}}z\right).$$

In this expression, *a* and *b* are respectively the shape and scale parameters of the gamma distribution (a>0, b>0), whereas m>0 is the so-called Nakagami parameter. $K_{a-m}$ stands for the modified Bessel function of order (a−m). The shape and scale parameters can be formulated in terms of physical parameters [22]:

(9) $$a=\frac{1}{e^{\frac{\sigma_{\Psi dB}^{2}}{\zeta^{2}}}-1};$$

(10) $$b=e^{\frac{\mu_{\Psi dB}}{\zeta }+\frac{\sigma_{\Psi dB}^{2}}{2\zeta^{2}}}\left(e^{\frac{\sigma_{\Psi dB}^{2}}{\zeta^{2}}}-1\right).$$

In these expressions, $\zeta =\frac{10}{\ln10}$, and $\mu_{\Psi dB}$ and $\sigma_{\Psi dB}$ are, respectively, the average and standard deviation of $\Psi dB=10\log_{10}\Psi =10\log_{10}\frac{P_{r}}{P_{t}}$, that is, the ratio of power received to power transmitted expressed in dB. The expected value $\mu_{\Psi dB}$ is given by the following expression [20]:

(11) $$\mu_{\Psi dB}=10\log_{10}\alpha -10\beta \log_{10}\frac{d}{d_{0}}.$$

Here, *d* is the distance between the transmitter and the receiver (transmission distance), $d_{0}$ is the reference distance, β is the path-loss exponent, and α is a constant that depends on multiple parameters (antenna gains, reference distance, path-loss exponent, average blockage, and carrier frequency). There is no mathematical expression for $\sigma_{\Psi dB}$ (shadowing spread), but its value has been experimentally set up within the range $\left[4,13\right]$ dB [20]. The generic path-loss model given in (11) can be replaced by more specific models (free-space, two-ray, Okumura, Hata, COST 231 models, etc.) when further details about the scenario are provided [20].

Equations (8)–(11) define a versatile propagation model that is entirely formulated in terms of physical (and measurable) parameters. To summarize, the inputs to this model are the constant α, the path-loss exponent, both the transmission and reference distances, the delay spread and the Nakagami parameter. This parameter can take on any value above 0, thus providing high flexibility to capture quite different small-scale multipath fading conditions:

- If m>1, the channel is Rician, meaning that there is a dominant LOS propagation component over the scattered non-LOS component. This is the so-called non-isotropic propagation environment. In this case, the degree of fading is low, becoming less severe with increasing *m*. In particular, for m→∞ there is no fading.
- If m≤1, there is no dominant LOS component in the received signal and the degree of fading is high, increasing as *m* decreases. Such scenario is referred to as isotropic propagation environment. Particular cases are m=1 (Rayleigh channel), m=0.5 (one-sided Gaussian channel) and m≪1 (very severe fading channel).

## 4. Average Energy Harvested

The first step in the characterization of the amount of energy harvested as a random variable is to obtain its average. Recalling Equation (7), the expected energy harvested can be formulated in this way:

(12) $$E\left\{E(T)\right\}=E\left\{\eta \int_{T}r^{2}\left(t\right)\cdot dt\right\}=\eta \int_{T}E\left\{r^{2}\left(t\right)\right\}\cdot dt=\frac{\eta }{2}\int_{T}E\left\{∥\tilde{r}{\left(t\right)∥}^{2}\right\}\cdot dt.$$

On the other hand, from Equation (7), we have

(13) $$\tilde{r}\left(t\right)=\left({\tilde{x}}_{I}\left(t\right)+j\cdot {\tilde{x}}_{Q}\left(t\right)\right)\cdot \left(h_{re}\left(t\right)+j\cdot h_{im}\left(t\right)\right)+{\tilde{n}}_{I}\left(t\right)+j\cdot {\tilde{n}}_{Q}\left(t\right).$$

In this expression, both $\tilde{x}\left(t\right)$ and $\tilde{n}\left(t\right)$ have been decomposed into their in-phase and quadrature components, respectively $\left({\tilde{x}}_{I}\left(t\right),{\tilde{x}}_{Q}\left(t\right)\right)$ and $\left({\tilde{n}}_{I}\left(t\right),{\tilde{n}}_{Q}\left(t\right)\right)$, and the channel coefficient into its real and imaginary parts, namely $\left(h_{re}\left(t\right),h_{im}\left(t\right)\right)$. Further manipulation allows to separate the real and imaginary components of $\tilde{r}\left(t\right)$ as follows:

(14) $$\tilde{r}\left(t\right)={\tilde{x}}_{I}\left(t\right)\cdot h_{re}\left(t\right)-{\tilde{x}}_{Q}\left(t\right)\cdot h_{im}\left(t\right)+{\tilde{n}}_{I}\left(t\right)+j\cdot \left({\tilde{x}}_{I}\left(t\right)\cdot h_{im}\left(t\right)+{\tilde{x}}_{Q}\left(t\right)\cdot h_{re}\left(t\right)+{\tilde{n}}_{Q}\left(t\right)\right).$$

Then, the squared module of $\tilde{r}\left(t\right)$ is nothing else but the sum of the squared real and imaginary parts:

(15) $$∥\tilde{r}{\left(t\right)∥}^{2}={\left({\tilde{x}}_{I}\left(t\right)\cdot h_{re}\left(t\right)-{\tilde{x}}_{Q}\left(t\right)\cdot h_{im}\left(t\right)+{\tilde{n}}_{I}\left(t\right)\right)}^{2}+{\left({\tilde{x}}_{I}\left(t\right)\cdot h_{im}\left(t\right)+{\tilde{x}}_{Q}\left(t\right)\cdot h_{re}\left(t\right)+{\tilde{n}}_{Q}\left(t\right)\right)}^{2}.$$

Proceeding through standard calculations, we can end up with the following exact result for $∥\tilde{r}{\left(t\right)∥}^{2}$:

(16) $$\begin{array}{c} ∥\tilde{r}{\left(t\right)∥}^{2}=∥\tilde{x}{\left(t\right)∥}^{2}\cdot {∥h\left(t\right)∥}^{2}+{\tilde{n}}_{I}^{2}\left(t\right)+{\tilde{n}}_{Q}^{2}\left(t\right)+2\left({\tilde{x}}_{I}\left(t\right)\cdot h_{re}\left(t\right)-{\tilde{x}}_{Q}\left(t\right)\cdot h_{im}\left(t\right)\right)\cdot {\tilde{n}}_{I}\left(t\right)\\ +2\left({\tilde{x}}_{I}\left(t\right)\cdot h_{im}\left(t\right)+{\tilde{x}}_{Q}\left(t\right)\cdot h_{re}\left(t\right)\right)\cdot {\tilde{n}}_{Q}\left(t\right). \end{array}$$

Next, we can obtain the expectation of $∥\tilde{r}{\left(t\right)∥}^{2}$. The analysis can be simplified by recalling some valuable properties of AWGN ([23]): (*i*) $E\left\{{\tilde{n}}_{I}^{2}\left(t\right)\right\}=E\left\{{\tilde{n}}_{Q}^{2}\left(t\right)\right\}=E\left\{n^{2}\left(t\right)\right\}=N_{R}=\eta_{0}\cdot B$, where $N_{R}$ stands for the received noise power, $\eta_{0}$ for the spectral density power and *B* for the power transfer bandwidth, (*ii*) $E\left\{{\tilde{n}}_{I}\left(t\right)\right\}=E\left\{{\tilde{n}}_{Q}\left(t\right)\right\}=0$, and (*iii*) ${\tilde{n}}_{I}\left(t\right)$ and ${\tilde{n}}_{Q}\left(t\right)$ are mutually independent Gaussian random variables. In addition, because noise is independent of both, input signal and channel coefficient, and $E\left\{{\tilde{n}}_{I}\left(t\right)\right\}=0$, we also have $E\left\{\left({\tilde{x}}_{I}\left(t\right)\cdot h_{re}\left(t\right)-{\tilde{x}}_{Q}\left(t\right)\cdot h_{im}\left(t\right)\right)\cdot {\tilde{n}}_{I}\left(t\right)\right\}=E\left\{\left({\tilde{x}}_{I}\left(t\right)\cdot h_{re}\left(t\right)-{\tilde{x}}_{Q}\left(t\right)\cdot h_{im}\left(t\right)\right)\right\}\cdot E\left\{{\tilde{n}}_{I}\left(t\right)\right\}=0$. Similarly, $E\left\{\left({\tilde{x}}_{I}\left(t\right)\cdot h_{im}\left(t\right)+{\tilde{x}}_{Q}\left(t\right)\cdot h_{re}\left(t\right)\right)\cdot {\tilde{n}}_{Q}\left(t\right)\right\}=0$. Because the input signal and the channel coefficient are also mutually independent random variables, the squared module of the complex envelope can be expressed in this way:

(17) $$E\left\{∥\tilde{r}{\left(t\right)∥}^{2}\right\}=E\left\{∥\tilde{x}{\left(t\right)∥}^{2}\right\}E\left\{{∥h\left(t\right)∥}^{2}\right\}+2E\left\{n^{2}\left(t\right)\right\}.$$

Accordingly, the average energy harvested formulated in (12) obeys the following expression:

(18) $$E\left\{E(T)\right\}=\eta \int_{T}\left(\frac{E\left\{∥\tilde{x}{\left(t\right)∥}^{2}\right\}}{2}E\left\{{∥h\left(t\right)∥}^{2}\right\}+E\left\{n^{2}\left(t\right)\right\}\right)\cdot dt.$$

The equivalent low-pass input signal $\tilde{x}\left(t\right)$ is defined by the modulation. Consequently, in general, this signal is non-stationary and can be expressed as $\tilde{x}\left(t\right)=A\left(t\right)e^{j\theta (t)}$, where A(t) and θ(t) denote, respectively, the time-varying amplitude and phase of the modulating signal. On the other hand, the channel is assumed to be stationary, and hence its effect represented by $E\left\{{∥h\left(t\right)∥}^{2}\right\}$ can be taken out of the integral in the previous equation. In [22], exact closed-form expressions are provided for the moments of the generalized-K distribution, among which the second moment is given by $E\left\{{∥h\left(t\right)∥}^{2}\right\}=a\cdot b$, where *a* and *b* obey, respectively, expressions (9) and (10). In agreement with [24], this second moment will be renamed from now on as $\Omega_{p}$. Accordingly, we have

(19) $$E\left\{E(T)\right\}=\eta \left(\Omega_{p}\int_{T}\frac{E\left\{A^{2}\left(t\right)\right\}}{2}\cdot dt+N_{R}\cdot T\right).$$

In this equation, the relevant term regarding how efficient the energy-transfer process can be is $\Omega_{p}$, because it represents the global channel effect due to path loss, shadowing and multi-path fading on the received signal. Instead, the integral in Equation (19) evaluates the energy contained in an interval *T* of the transmitted signal, whatever its shape. Thus, without loss of generality, we can assume the simplest case of A(t)=A, which corresponds to an unmodulated carrier. Accordingly, the previous equation can be reformulated in this way:

(20) $$E\left\{E(T)\right\}=\eta \cdot T\left(\frac{A^{2}}{2}\cdot \Omega_{p}+N_{R}\right).$$

The term $\frac{A^{2}}{2}$ is nothing else but the transmitted power (normalized to a 1Ω-load). If we denote this power as $P_{t}$, the expected energy harvested is as follows:

(21) $$E\left\{E(T)\right\}=\eta \cdot T\left(P_{t}\cdot \Omega_{p}+N_{R}\right).$$

Note that the average energy harvested depends on the shape and scale parameters of the gamma distribution (via $\Omega_{p}$), but not on the Nakagami parameter that characterizes multi-path fading. Note also that it has two components, the main one due to the power-bearing signal, and the thermal noise.

## 5. Variance of Energy Harvested

To obtain the variance of the energy harvested, first we can analyze the second moment about zero:

(22) $$\begin{array}{cc} E\left\{E^{2}\left(T\right)\right\} & =\eta^{2}E\left\{{\left(\int_{T}r^{2}\left(t\right)dt\right)}^{2}\right\}=\eta^{2}E\left\{\left(\int_{T}r^{2}\left(s\right)ds\right)\left(\int_{T}r^{2}\left(t\right)dt\right)\right\}\\ \; & =\eta^{2}E\left\{\int_{T}\int_{T}r^{2}\left(s\right)r^{2}\left(t\right)ds\cdot dt\right\}=\eta^{2}\int_{T}\int_{T}E\left\{r^{2}\left(s\right)r^{2}\left(t\right)\right\}ds\cdot dt\\ \; & =\frac{\eta^{2}}{4}\int_{T}\int_{T}E\left\{∥\tilde{r}{\left(s\right)∥}^{2}{∥\tilde{r}\left(t\right)∥}^{2}\right\}ds\cdot dt. \end{array}$$

The next step is to evaluate the expectation inside the integral, which is nothing else but a correlation. However, the analytical procedure that yields an exact closed-form expression for this correlation is very complex, and thus the details have been relegated to the Appendix A. For the setting s=t+τ, the result is Equation (A31):

(23) $$\begin{array}{cc} E\left\{∥\tilde{r}{\left(t+\tau \right)∥}^{2}{∥\tilde{r}\left(t\right)∥}^{2}\right\} & =\phi_{∥\tilde{r}{∥}^{2}}\left(t,\tau \right)=\phi_{∥\tilde{x}{∥}^{2}}\left(t,\tau \right)\phi_{{∥h∥}^{2}}\left(\tau \right)+4N_{R}\cdot E\left\{∥\tilde{x}{\left(t\right)∥}^{2}\right\}E\left\{{∥h\left(t\right)∥}^{2}\right\}\\ \; & +16\phi_{\tilde{n}}\left(\tau \right)\cdot ℜ\left\{\phi_{\tilde{x}}\left(t,\tau \right)\phi_{h}\left(\tau \right)\right\}+4N_{R}^{2}+4\phi_{\tilde{n}}^{2}\left(\tau \right). \end{array}$$

Next, the terms that appear in this equation are analyzed individually.

If we assume that the input signal is an unmodulated carrier, that is, $\tilde{x}\left(t\right)=Ae^{j\theta }$, we have

(24) $$E\left\{∥\tilde{x}{\left(t\right)∥}^{2}\right\}=A^{2}=2P_{t};$$

(25) $$\phi_{∥\tilde{x}{∥}^{2}}\left(t,\tau \right)=E\left\{∥\tilde{x}{\left(t+\tau \right)∥}^{2}{∥\tilde{x}\left(t\right)∥}^{2}\right\}=A^{4}=4P_{t}^{2};$$

(26) $$\phi_{\tilde{x}}\left(t,\tau \right)=\frac{1}{2}E\left\{\tilde{x}\left(t+\tau \right){\tilde{x}}^{*}\left(t\right)\right\}=\frac{1}{2}E\left\{Ae^{j\theta }\cdot Ae^{-j\theta }\right\}=\frac{A^{2}}{2}=P_{t}.$$

Note that, for the case of an unmodulated carrier, the input signal does not only become a stationary process, but its associated correlations are constant. Moreover, despite the fact that it has been assumed that θ is a constant phase offset, the analysis that follows would also be valid, with some minor modifications, for a phase-modulated signal, that is, θ=θ(t).

Another auto-correlation involved in (23) is $\phi_{\tilde{n}}\left(\tau \right)$. For the case of AWGN, the following expression is provided in [23]:

(27) $$\phi_{\tilde{n}}\left(\tau \right)=\eta_{0}\frac{\sin\left(\pi B\tau \right)}{\pi \tau }=\eta_{0}B\frac{\sin\left(\pi B\tau \right)}{\pi B\tau }=N_{R}\cdot \mathrm{sinc}\left(\pi B\tau \right).$$

The remaining terms are $\phi_{h}\left(\tau \right)$ and $\phi_{{∥h∥}^{2}}\left(\tau \right)$, which are channel auto-correlations. They capture the variations, in the statistical sense, perceived by the user as it moves at a certain speed *v* over the combined path loss, shadowing, and multi-path fading scenario under consideration (in the present case, the scenario that leads to the generalized-K distribution). These temporal correlations can always be transformed into spatial correlations, because the dependence on τ is, in fact, on the product v·τ. However, because the focus of this paper is on static users, that is, v=0, which has the same effect as τ=0, we are really interested in $\phi_{h}\left(0\right)$ and $\phi_{{∥h∥}^{2}}\left(0\right)$. Regarding the first one, we can write

(28) $$\phi_{h}\left(0\right)=\frac{1}{2}E\left\{h\left(t\right)\cdot h^{*}\left(t\right)\right\}=\frac{1}{2}E\left\{{∥h\left(t\right)∥}^{2}\right\}=\frac{\Omega_{p}}{2}.$$

To analyze the second term, it is useful to introduce the auto-covariance $\mu_{{∥h∥}^{2}}\left(\tau \right)$, which is related to the auto-correlation as follows:

(29) $$\mu_{{∥h∥}^{2}}\left(\tau \right)=\phi_{{∥h∥}^{2}}\left(\tau \right)-E\left\{{∥h\left(t+\tau \right)∥}^{2}\right\}\cdot E\left\{{∥h\left(t\right)∥}^{2}\right\}.$$

Accordingly,

(30) $$\phi_{{∥h∥}^{2}}\left(0\right)=\mu_{{∥h∥}^{2}}\left(0\right)+E^{2}\left\{{∥h\left(t\right)∥}^{2}\right\}=\mu_{{∥h∥}^{2}}\left(0\right)+\Omega_{p}^{2}.$$

Because the auto-covariance at the origin is nothing else but the variance, we have

(31) $$\phi_{{∥h∥}^{2}}\left(0\right)=Var\left\{{∥h\left(t\right)∥}^{2}\right\}+\Omega_{p}^{2}.$$

Moreover, $Var\left\{{∥h\left(t\right)∥}^{2}\right\}$ can be expressed in terms of the second and fourth moments of the distribution of ∥h(t)∥:

(32) $$Var\left\{{∥h\left(t\right)∥}^{2}\right\}=E\left\{{∥h\left(t\right)∥}^{4}\right\}-E^{2}\left\{{∥h\left(t\right)∥}^{2}\right\}.$$

Note that the second term in the right-hand side of this equation is $\Omega_{p}^{2}$. In [22], a generic closed-form expression is provided for the moments of the generalized-K distribution. This expression is exact and can be particularized for the fourth moment as follows:

(33) $$E\left\{{∥h\left(t\right)∥}^{4}\right\}=\frac{\Gamma (a+2)\Gamma (m+2)}{\Gamma (a)\Gamma (m)}{\left(\frac{b}{m}\right)}^{2}.$$

Recall that *a* and *b* are, respectively, the scale and shape parameters of the gamma distribution that describes path loss and shadowing, and *m* is the Nakagami parameter that characterizes multi-path fading. Next, considering Equations (32) and (33), Equation (31) can be rewritten in terms of the parameters of the generalized-K distribution:

(34) $$\phi_{{∥h∥}^{2}}\left(0\right)=\frac{\Gamma (a+2)\Gamma (m+2)}{\Gamma (a)\Gamma (m)}{\left(\frac{b}{m}\right)}^{2}.$$

Now, introducing (24)–(28) and (34) into Equation (23), and rearranging terms, we can obtain the definite result for $E\left\{∥\tilde{r}{\left(t+\tau \right)∥}^{2}{∥\tilde{r}\left(t\right)∥}^{2}\right\}$:

(35) $$\begin{array}{cc} E\left\{∥\tilde{r}{\left(t+\tau \right)∥}^{2}{∥\tilde{r}\left(t\right)∥}^{2}\right\} & =4P_{t}^{2}\frac{\Gamma (a+2)\Gamma (m+2)}{\Gamma (a)\Gamma (m)}{\left(\frac{b}{m}\right)}^{2}\\ \; & +8N_{R}P_{t}\Omega_{p}\left(1+\mathrm{sinc}\left(\pi B\tau \right)\right)\\ \; & +4N_{R}^{2}\left(1+{\mathrm{sinc}}^{2}\left(\pi B\tau \right)\right). \end{array}$$

As can be seen, this expression depends exclusively on system parameters. In particular, $P_{t}\Omega_{p}$ is the average received power, because it is the product of the average transmitted power and the channel effect represented by $\Omega_{p}$. Finally, the variance of the energy harvested can be obtained by integrating expression (35) according to (22), and then subtracting the square of the expected energy harvested given by (21). An exact closed-form expression is obtained:

(36) $$\begin{array}{c} \begin{array}{cc} Var\left\{E(T)\right\} & ={\left(\eta P_{t}T\right)}^{2}\left(\frac{\Gamma (a+2)\Gamma (m+2)}{\Gamma (a)\Gamma (m)}{\left(\frac{b}{m}\right)}^{2}-\Omega_{p}^{2}\right)\\ & +\frac{4\eta^{2}N_{R}P_{t}\Omega_{p}}{\pi^{2}B^{2}}\left(-1+\cos\left(\pi BT\right)+\pi BT\cdot Si\left(\pi BT\right)\right)\\ & +\frac{\eta^{2}N_{R}^{2}}{\pi^{2}B^{2}}(-1-\gamma +\cos\left(2\pi BT\right)+Ci\left(2\pi BT\right)\\ & -\ln(2\pi BT)+2\pi BT\cdot Si(2\pi BT)). \end{array} \end{array}$$

Here, γ is the Euler’s constant (γ≃0.577216), and Si() and Ci() stand, respectively, for the sine integral and cosine integral functions, which are defined as follows:

(37) $$Si\left(x\right)=\int_{0}^{x}\frac{\sin(u)}{u}du;$$

(38) $$Ci\left(x\right)=-\int_{x}^{\infty }\frac{\cos(u)}{u}du.$$

As can be seen from Equation (36), the variance of the energy harvested has three components: one that depends exclusively on the power-bearing signal, a cross-term that depends on both the power-bearing signal and the thermal noise, and finally a third component that only depends on noise.

Even more meaningful than the variance is the squared coefficient of variation, which is nothing more than the ratio of the variance to the squared expectation. In essence, it represents the relative variability of the distribution around its expected value. In particular, the squared coefficient of variation of the energy harvested, namely $SCV\left\{E(T)\right\}$, is given by

(39) $$\begin{array}{c} \begin{array}{cc} SCV\left\{E(T)\right\} & =P_{t}^{2}\frac{\left(\frac{\Gamma (a+2)\Gamma (m+2)}{\Gamma (a)\Gamma (m)}{\left(\frac{b}{m}\right)}^{2}-\Omega_{p}^{2}\right)}{{\left(P_{t}\Omega_{p}+N_{R}\right)}^{2}}\\ & +\frac{4N_{R}P_{t}\Omega_{p}\left(-1+\cos\left(\pi BT\right)+\pi BT\cdot Si\left(\pi BT\right)\right)}{\pi^{2}B^{2}T^{2}{\left(P_{t}\Omega_{p}+N_{R}\right)}^{2}}\\ & +\frac{N_{R}^{2}}{\pi^{2}B^{2}T^{2}{\left(P_{t}\Omega_{p}+N_{R}\right)}^{2}}(-1-\gamma +\cos\left(2\pi BT\right)\\ & +Ci(2\pi BT)-\ln(2\pi BT)+2\pi BT\cdot Si(2\pi BT)). \end{array} \end{array}$$

If the signal-to-noise ratio is very high, we can approximate the squared coefficient of variation of the energy harvested ($SCV\left\{E(T)\right\}$) by the next limit:

(40) $$\lim_{N_{R}\to 0}SCV\left\{E(T)\right\}=\frac{\Gamma (a+2)\Gamma (m+2)}{\Gamma \left(a\right)\Gamma \left(m\right)\Omega_{p}^{2}}{\left(\frac{b}{m}\right)}^{2}-1.$$

Recall that $\Omega_{p}=a\cdot b$. Equation (40) is nothing else but the squared coefficient of variation of ${∥h\left(t\right)∥}^{2}$.

## 6. Validation and Performance Assessment

In this section, the analysis performed in this paper is validated via simulation, and then the evolution of $E\left\{E(T)\right\}$ and $SCV\left\{E(T)\right\}$ in terms of multiple input variables is studied. With no loss of generality, an exposition time of 1 minute is assumed. To highlight the possibilities of the RF energy harvesting technology, a long-range scenario is considered, which is based on a real case: the KING-TV tower located at Seattle (Washington, DC, USA). This telecommunications tower transmits several analog and digital TV channels in the VHF and UHF bands, respectively. In particular, it uses a source power of 960 kW to broadcast a 6-MHz digital TV signal at the frequency of 0.677 GHz. The evaluation that follows assumes that all transmit power is concentrated on this frequency ($f_{p}=0.677$ GHz), though the receiver bandwidth is kept to 6 MHz (B=6 MHz). Other fixed parameters are the reference distance ($d_{0}=1$ m), the energy conversion efficiency (η=0.5), the ambient temperature (290 K), and the receiver noise figure (9 dB).

For the simulation and performance assessment, four input variables were taken into consideration: the transmission distance, the path-loss exponent, the shadowing spread, and the Nakagami parameter. Accordingly, Table 2 shows the analytical and simulation results for both the expected and squared coefficient of variation of the energy harvested. For each set of values, at least 30,000 runs were executed in order to achieve relative errors within 10% at a 90% confidence level. The relative errors between the analytical and simulation results have also been added to the table. As can be seen, there is a high agreement between the two sets of results.

**Figure 6:**
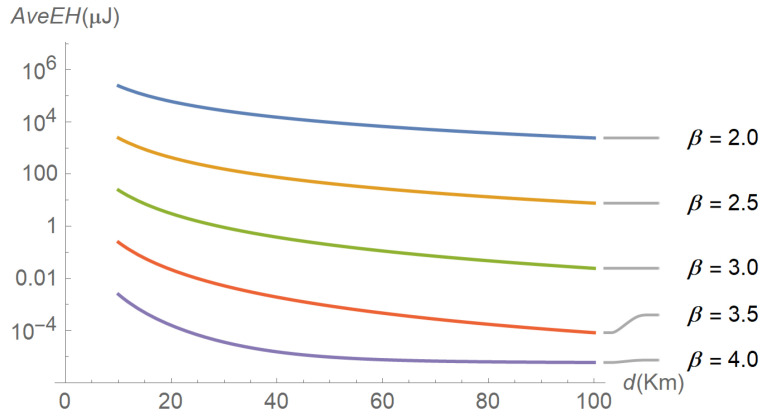
Evolution of the average energy harvested as a function of distance, for different path-loss exponents and a shadowing spread of 8.5 dB.

**Figure 7:**
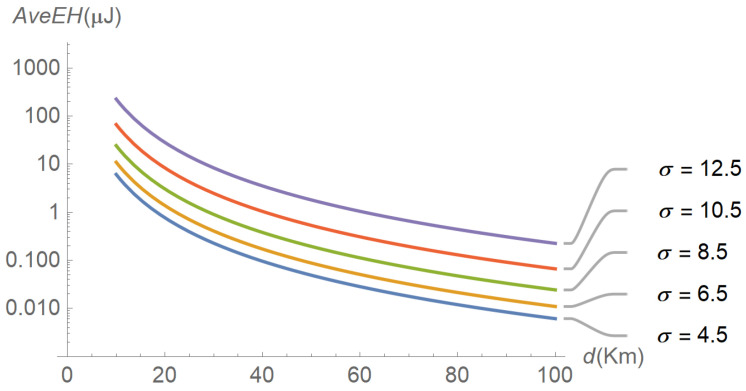
Evolution of the average energy harvested as a function of distance, for different levels of shadowing spread and a path-loss exponent equal to 3.0.

**Figure 8:**
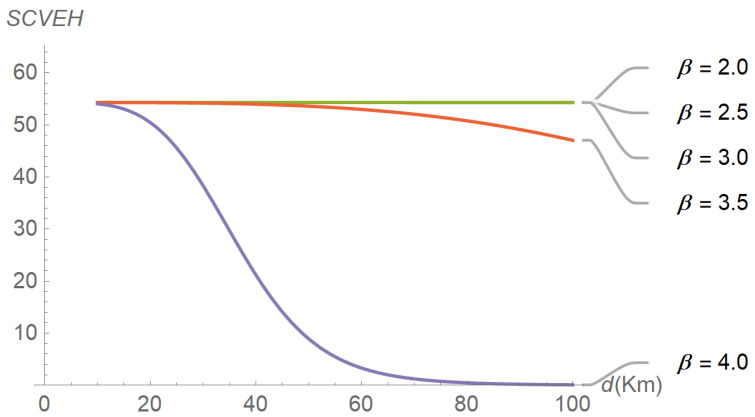
Evolution of the squared coefficient of variation in terms of distance, for different path-loss exponents. The shadowing spread and the Nakagami parameter have been set to 8.5 and 5.0, respectively.

**Figure 9:**
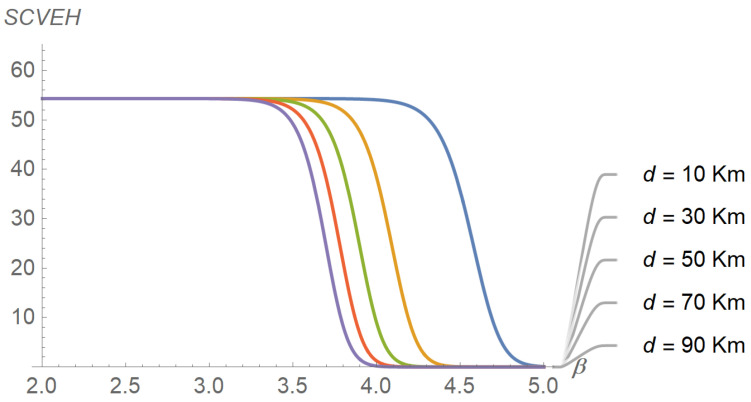
Evolution of the squared coefficient of variation in terms of the path-loss exponent, for different distances. The shadowing spread and the Nakagami parameter have been set to 8.5 and 5.0, respectively. The outermost curve corresponds to *d* = 10,000 m.

**Figure 10:**
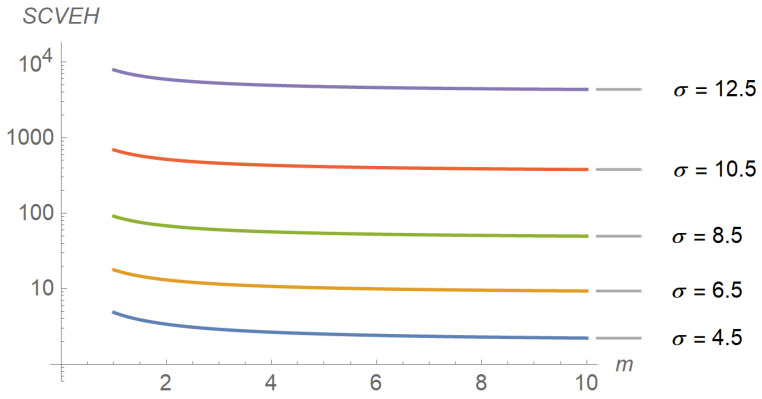
Evolution of the squared coefficient of variation as a function of the Nakagami parameter, for different values of the shadowing spread. The transmission distance and the path-loss exponent have been set to 50,000 m. and 3.0, respectively.

To assess the performance of the energy harvesting process, several input–output relations were explored, the results of which are reflected in subsequent figures. For instance, Figure 6 plots the evolution of the average energy harvested in terms of distance for different values of the path-loss exponent. As expected, the average energy harvested increases as the distance and the path-loss exponent decrease. A similar plot is shown in Figure 7, but parameterized by the shadowing spread instead of the path-loss exponent. The figure reveals that the average energy harvested increases with the shadowing spread. The interpretation is less intuitive, but we can think of shadowing as a low-frequency “noise” superimposed on the signal, the power of which is directly proportional to its variability (as occurs with thermal noise). Figure 8, Figure 9 and Figure 10 describe the behavior of the squared coefficient of variation. In particular, Figure 8 shows the dependence of this coefficient on distance, for different path-loss exponents. We can observe that the squared coefficient of variation decreases as the distance and/or the path-loss exponent increase, that is, as the expected energy harvested decreases. Such a reduction of variability with the decrease of the average is typical of non-negative random variables, like the energy harvested considered here. Figure 8 does not allow us to distinguish between the curves obtained for the lowest path-loss exponents. However, these differences can be better highlighted by exchanging the roles of distance and path-loss exponent in the representation. This is shown in Figure 9, which confirms that beyond β≅3.5 the decay profiles begin to distinguish. Finally, Figure 10 shows how the squared coefficient of variation varies with the shadowing spread and the Nakagami parameter. As can be seen, the influence of the shadowing spread is much higher than that of the Nakagami parameter. The figure also highlights the fact that the squared coefficient of variation of the energy harvested can vary within a very large range, consistent with the relatively shorter variability of the shadowing spread.

## 7. Discussion

In this paper, exact closed-form expressions for the mean and variance (and squared coefficient of variation) of the energy harvested by a static device have been obtained and validated via simulation. To model the propagation scenario, the generalized-K model has been adopted, as it encompasses the effects of path-loss, shadowing and multi-path fading for a wide set of wireless scenarios. It has been assumed that the device is illuminated for an arbitrary exposure time by a dedicated source emitting an unmodulated carrier. A long-range scenario has also been assumed, in order to highlight the capabilities of the RF energy-harvesting technology.

The results obtained in this paper reveal that the path-loss and shadowing components of the propagation model have a much greater influence on the amount of energy harvested than the multi-path component. They also reveal that the squared coefficient of variation of the harvested energy can be very large. This is in agreement with the high variability that the signal level can experience in a generalized-K propagation environment. Consequently, the RF-EH device must be designed to operate under wide dynamic ranges at its input, which in turn means a very low sensitivity threshold and high saturation point.

Future work can go in several directions. The following list includes some suggestions.

- As stated in Section 1, the results obtained in this paper can be used to determine relevant metrics in the context of future WENs. Examples of these metrics are the probability of energy outage and the mean waiting time experienced by energy requests.
- Also, this work can be extended to the case of mobile devices and to the evaluation of higher-order moments of the harvested energy.
- The RF energy harvester considered in this paper is ideal. However, a more realistic model of such device should include undesirable phenomena, such as limited sensitivity, saturation effects and non-linearities. Therefore, another extension could consist of evaluating the impact of these perturbations on the energy-harvesting process.
- Finally, to reinforce the results derived from this work, an experimental validation is required.

## Figures and Tables

**Table 1:** Current state-of-the-art work in RF-EH involving channel propagation models.

Reference	Propagation Model	Main Focus
[9]	Free space path-loss and additive Gaussian noise	Fair energy allocation
[10]	Two-ray/Path-loss, lognormal shadowing and Rician fading	Average energy harvested/Estimation of model parameters
[11]	Path-loss, Rayleigh fading and additive Gaussian noise	Beamforming strategy
[12]	Nakagami-m fading	Optimal transmission policy
[13]	Empirical	Theoretical bounds on transmission rate
[14]	Empirical	Design guidelines for RF-to-DC circuitry
[15]	Nakagsami-m/Generalized-K	Battery recharging time
[16]	Empirical (HATA/Ericsson/ITU-R)	Spectral behavior of RF-EH in suburban environments
[17]	Gaussian	Mean and variance of harvested energy (from multiple transmitters)
[18]	Generalized η-μ/κ-μ	Battery recharging time
Current contribution	Generalized-K	Mean and variance of harvested energy

**Table 2:** Comparison between analytical and simulation results for different parameter sets. The relative error (in absolute value) incurred by the simulation results has also been included.

*d*(m), β, $\sigma_{\Psi \mathrm{dB}}$, *m*	$E\left\{E(T)\right\}$ Analytical	$E\left\{E(T)\right\}$ Simulation
	$\mathrm{SCV}\left\{E(T)\right\}$ Analytical	$\mathrm{SCV}\left\{E(T)\right\}$ Simulation
10,000, 3.0, 8.5, 2.0	24.3135	25.1643 (3.5%)
	68.1367	68.2532 (0.2%)
10,000, 2.0, 5.5, 0.3	79,855.3	77,096.3 (3.5%)
	20.5454	21.0171 (2.3%)
20,000, 2.0, 6.5, 4.0	27,440.9	26,991.4 (1.6%)
	10.7423	10.0688 (6.3%)
20,000, 3.0, 8.5, 5.0	3.03919	2.9721 (2.2%)
	54.3092	54.1187 (0.4%)
30,000, 3.5, 6.5, 7.0	0.00235284	0.00233108 (0.9%)
	9.6885	9.19882 (5.1%)
30,000, 2.5, 4.5, 9.0	39.2981	39.7297 (1.1%)
	2.2511	2.24226 (0.4%)
40,000, 4.0, 8.5, 6.0	0.0000152195	0.0000151148 (0.7%)
	20.5507	19.1669 (6.7%)
40,000, 2.0, 6.5, 0.5	6860.24	6720.51 (2.0%)
	27.1815	24.6008 (9.5%)
50,000, 2.5, 4.5, 3.0	10.9585	11.0875 (1.2%)
	2.90132	2.7808 (4.2%)
50,000, 3.5, 10.5, 8.0	0.00238773	0.00235364 (1.4%)
	385.967	361.542 (6.3%)
60,000, 3.0, 8.0, 5.0	0.0904563	0.0897801 (0.7%)
	34.7094	33.9685 (2.1%)
60,000, 2.0, 4.0, 10.0	1520.35	1529.88 (0.6%)
	1.56925	1.551 (1.2%)
70,000, 2.5, 6.0, 1.0	7.17395	7.23829 (0.9%)
	12.4884	12.2202 (2.1%)
70,000, 2.5, 6.0, 0.1	7.17395	6.77048 (5.6%)
	73.1861	77.2072 (5.5%)
80,000, 4.0, 10.0, 3.0	$6.96074\times 10^{-6}$	$6.95204\times 10^{-6}$ (0.1%)
	8.44362	8.39472 (0.6%)
80,000, 3.0, 7.0, 7.0	0.0256447	0.0253276 (1.2%)
	14.3489	13.7613 (4.1%)
90,000, 2.5, 10.5, 6.0	27.3988	27.1551 (0.9%)
	402.224	407.723 (1.4%)
90,000, 3.5, 8.0, 1.0	0.0000950559	0.0000937424 (1.4%)
	51.6893	50.6754 (2.0%)
100,000, 2.0, 7.5, 4.0	1590.89	1523.16 (4.3%)
	23.6669	23.0866 (2.5%)
100,000, 3.0, 4.5, 0.7	0.00613169	0.0061943 (1.0%)
	6.0946	6.17654 (1.3%)

## Data Availability

Not applicable.

## Abbreviations

The following abbreviations are used in this manuscript:

5G/6G	Fifth Generation/Sixth Generation
AWGN	Additive White Gaussian Noise
CSI	Channel State Information
EH	Energy Harvesting
IoT	Internet of Things
LOS	Line of Sight
NLOS	Non Line of Sight
RF-EH	Radio Frequency-based Energy Harvesting
SWIPT	Simultaneous Wireless Information and Power Transfer
UAV	Unmanned Autonomous Vehicle
WEN	Wireless Energy Network
WIPT	Wireless Information and Power Transfer
WIT	Wireless Information Transfer
WPT	Wireless Power Transfer

## Appendix A

As stated in Section 5, the variance of the energy harvested is based on the expectation $E\left\{∥\tilde{r}{\left(s\right)∥}^{2}{∥\tilde{r}\left(t\right)∥}^{2}\right\}$. By recalling Equation (16), the argument of such expectation can be formulated as follows:

(A1) $$\begin{array}{cc} \; & ∥\tilde{r}{\left(s\right)∥}^{2}∥\tilde{r}{\left(t\right)∥}^{2}=(∥\tilde{x}{\left(s\right)∥}^{2}{∥h\left(s\right)∥}^{2}+{\tilde{n}}_{I}^{2}\left(s\right)+{\tilde{n}}_{Q}^{2}\left(s\right)\\ \; & +2\left({\tilde{x}}_{I}\left(s\right)\cdot h_{re}\left(s\right)-{\tilde{x}}_{Q}\left(s\right)\cdot h_{im}\left(s\right)\right)\cdot {\tilde{n}}_{I}\left(s\right)+2\left({\tilde{x}}_{I}\left(s\right)\cdot h_{im}\left(s\right)+{\tilde{x}}_{Q}\left(s\right)\cdot h_{re}\left(s\right)\right)\cdot {\tilde{n}}_{Q}\left(s\right))\\ \; & \cdot (∥\tilde{x}{\left(t\right)∥}^{2}{∥h\left(t\right)∥}^{2}+{\tilde{n}}_{I}^{2}\left(t\right)+{\tilde{n}}_{Q}^{2}\left(t\right)\\ \; & +2\left({\tilde{x}}_{I}\left(t\right)\cdot h_{re}\left(t\right)-{\tilde{x}}_{Q}\left(t\right)\cdot h_{im}\left(t\right)\right)\cdot {\tilde{n}}_{I}\left(t\right)+2\left({\tilde{x}}_{I}\left(t\right)\cdot h_{im}\left(t\right)+{\tilde{x}}_{Q}\left(t\right)\cdot h_{re}\left(t\right)\right)\cdot {\tilde{n}}_{Q}\left(t\right)). \end{array}$$

To evaluate the expected value of such a long expression, we can first identify the following terms:

- $I_{1}:∥\tilde{x}{\left(s\right)∥}^{2}{∥h\left(s\right)∥}^{2}$.
- $I_{2}:2\left({\tilde{x}}_{I}\left(s\right)\cdot h_{re}\left(s\right)-{\tilde{x}}_{Q}\left(s\right)\cdot h_{im}\left(s\right)\right)\cdot {\tilde{n}}_{I}\left(s\right)+2\left({\tilde{x}}_{I}\left(s\right)\cdot h_{im}\left(s\right)+{\tilde{x}}_{Q}\left(s\right)\cdot h_{re}\left(s\right)\right)\cdot {\tilde{n}}_{Q}\left(s\right)$.
- $I_{3}:{\tilde{n}}_{I}^{2}\left(s\right)+{\tilde{n}}_{Q}^{2}\left(s\right)$.
- $I_{4}:∥\tilde{x}{\left(t\right)∥}^{2}{∥h\left(t\right)∥}^{2}$.
- $I_{5}:2\left({\tilde{x}}_{I}\left(t\right)\cdot h_{re}\left(t\right)-{\tilde{x}}_{Q}\left(t\right)\cdot h_{im}\left(t\right)\right)\cdot {\tilde{n}}_{I}\left(t\right)+2\left({\tilde{x}}_{I}\left(t\right)\cdot h_{im}\left(t\right)+{\tilde{x}}_{Q}\left(t\right)\cdot h_{re}\left(t\right)\right)\cdot {\tilde{n}}_{Q}\left(t\right)$.
- $I_{6}:{\tilde{n}}_{I}^{2}\left(t\right)+{\tilde{n}}_{Q}^{2}\left(t\right)$.

Note that $I_{1}$, $I_{2}$ and $I_{3}$ are formally equivalent to $I_{4}$, $I_{5}$ and $I_{6}$, respectively. According to these new variables, Equation (A1) can be simply reformulated in the following way:

(A2) $$∥\tilde{r}{\left(s\right)∥}^{2}{∥\tilde{r}\left(t\right)∥}^{2}=\left(I_{1}+I_{2}+I_{3}\right)\cdot \left(I_{4}+I_{5}+I_{6}\right).$$

The subsequent procedure consists of developing all terms generated by the product in Equation (A2), and then take their respective expectations. In fact, only six out of the nine terms lead to different results:

Product $I_{1}\cdot I_{4}$

(A3)
$$I_{1}\cdot I_{4}=∥\tilde{x}{\left(s\right)∥}^{2}{∥h\left(s\right)∥}^{2}∥\tilde{x}{\left(t\right)∥}^{2}{∥h\left(t\right)∥}^{2}.$$

Then, the expected value can be obtained after several manipulations:

(A4) $$\begin{array}{c} \begin{array}{cc} E\left\{I_{1}\cdot I_{4}\right\} & =E\left\{∥\tilde{x}{\left(s\right)∥}^{2}{∥h\left(s\right)∥}^{2}∥\tilde{x}{\left(t\right)∥}^{2}{∥h\left(t\right)∥}^{2}\right\}\\ & =E\left\{∥\tilde{x}{\left(s\right)∥}^{2}{∥\tilde{x}\left(t\right)∥}^{2}\right\}E\left\{{∥h\left(s\right)∥}^{2}{∥h\left(t\right)∥}^{2}\right\}\\ & =E\left\{∥\tilde{x}{\left(t+\tau \right)∥}^{2}{∥\tilde{x}\left(t\right)∥}^{2}\right\}E\left\{{∥h\left(t+\tau \right)∥}^{2}{∥h\left(t\right)∥}^{2}\right\}\\ & =\phi_{∥\tilde{x}{∥}^{2}}\left(t,\tau \right)\cdot \phi_{{∥h∥}^{2}}\left(\tau \right). \end{array} \end{array}$$

Note that the total expectation has been expressed as a product of expectations thanks to the independence between $\tilde{x}\left(t\right)$ and h(t). Moreover, the temporal variable *s* has been replaced, with no loss of generality, by t+τ, where τ is a time lag. The first expectation is nothing else but the auto-correlation of the squared module of $\tilde{x}$ in terms of the absolute time and the time lag τ, because no assumption has yet been made about the input signal being stationary (it depends on the type of modulation). Instead, because the channel is assumed to be stationary, the second auto-correlation depends exclusively on the time lag.

Product $I_{1}\cdot I_{5}$

(A5)
$$\begin{array}{cc} I_{1}\cdot I_{5}=2∥\tilde{x}{\left(s\right)∥}^{2}{∥h\left(s\right)∥}^{2}\cdot (({\tilde{x}}_{I}\left(t\right)\cdot & h_{re}\left(t\right)-{\tilde{x}}_{Q}\left(t\right)\cdot h_{im}\left(t\right))\cdot {\tilde{n}}_{I}\left(t\right)\\ \; & +\left({\tilde{x}}_{I}\left(t\right)\cdot h_{im}\left(t\right)+{\tilde{x}}_{Q}\left(t\right)\cdot h_{re}\left(t\right)\right)\cdot {\tilde{n}}_{Q}\left(t\right)). \end{array}$$

Because noise is independent of both input signal and channel coefficient, the expected value of the previous expression depends directly on the expected values of the in-phase and quadrature components of noise, which are identically zero: $E\left\{{\tilde{n}}_{I}\left(t\right)\right\}=E\left\{{\tilde{n}}_{Q}\left(t\right)\right\}=0$. Accordingly,

(A6) $$E\left\{I_{1}\cdot I_{5}\right\}=0.$$

Product $I_{1}\cdot I_{6}$

(A7)
$$I_{1}\cdot I_{6}=∥\tilde{x}{\left(s\right)∥}^{2}{∥h\left(s\right)∥}^{2}\left({\tilde{n}}_{I}^{2}\left(t\right)+{\tilde{n}}_{Q}^{2}\left(t\right)\right).$$

Again because of the mutual independence between input signal and channel coefficient, we have

(A8) $$\begin{array}{c} \begin{array}{cc} E\left\{I_{1}\cdot I_{6}\right\} & =E\left\{∥\tilde{x}{\left(s\right)∥}^{2}\right\}E\left\{{∥h\left(s\right)∥}^{2}\right\}E\left\{\left({\tilde{n}}_{I}^{2}\left(t\right)+{\tilde{n}}_{Q}^{2}\left(t\right)\right)\right\}\\ & =E\left\{∥\tilde{x}{\left(s\right)∥}^{2}\right\}E\left\{{∥h\left(s\right)∥}^{2}\right\}\left(E\left\{{\tilde{n}}_{I}^{2}\left(t\right)\right\}+E\left\{{\tilde{n}}_{Q}^{2}\left(t\right)\right\}\right)\\ & =2N_{R}\cdot E\left\{∥\tilde{x}{\left(s\right)∥}^{2}\right\}E\left\{{∥h\left(s\right)∥}^{2}\right\}. \end{array} \end{array}$$

Here, the first property of AWGN stated in Section 4 has been recalled.

Product $I_{2}\cdot I_{4}$

This product is formally equivalent to $I_{1}\cdot I_{5}$; thus, we have

(A9) $$E\left\{I_{2}\cdot I_{4}\right\}=0.$$

Product $I_{2}\cdot I_{5}$

(A10)
$$\begin{array}{c} \begin{array}{cc} I_{2}\cdot I_{5} & =4(\left({\tilde{x}}_{I}\left(s\right)\cdot h_{re}\left(s\right)-{\tilde{x}}_{Q}\left(s\right)\cdot h_{im}\left(s\right)\right)\cdot {\tilde{n}}_{I}\left(s\right)\\ & \cdot \left({\tilde{x}}_{I}\left(t\right)\cdot h_{re}\left(t\right)-{\tilde{x}}_{Q}\left(t\right)\cdot h_{im}\left(t\right)\right)\cdot {\tilde{n}}_{I}\left(t\right)\\ & +\left({\tilde{x}}_{I}\left(s\right)\cdot h_{re}\left(s\right)-{\tilde{x}}_{Q}\left(s\right)\cdot h_{im}\left(s\right)\right)\cdot {\tilde{n}}_{I}\left(s\right)\\ & \cdot \left({\tilde{x}}_{I}\left(t\right)\cdot h_{im}\left(t\right)+{\tilde{x}}_{Q}\left(t\right)\cdot h_{re}\left(t\right)\right)\cdot {\tilde{n}}_{Q}\left(t\right)\\ & +\left({\tilde{x}}_{I}\left(s\right)\cdot h_{im}\left(s\right)+{\tilde{x}}_{Q}\left(s\right)\cdot h_{re}\left(s\right)\right)\cdot {\tilde{n}}_{Q}\left(s\right)\\ & \cdot \left({\tilde{x}}_{I}\left(t\right)\cdot h_{re}\left(t\right)-{\tilde{x}}_{Q}\left(t\right)\cdot h_{im}\left(t\right)\right)\cdot {\tilde{n}}_{I}\left(t\right)\\ & +\left({\tilde{x}}_{I}\left(s\right)\cdot h_{im}\left(s\right)+{\tilde{x}}_{Q}\left(s\right)\cdot h_{re}\left(s\right)\right)\cdot {\tilde{n}}_{Q}\left(s\right)\\ & \cdot \left({\tilde{x}}_{I}\left(t\right)\cdot h_{im}\left(t\right)+{\tilde{x}}_{Q}\left(t\right)\cdot h_{re}\left(t\right)\right)\cdot {\tilde{n}}_{Q}\left(t\right)). \end{array} \end{array}$$

This expression involves the following expectations:

(A11) $$\begin{array}{c} \begin{array}{cc} E\left\{{\tilde{n}}_{I}\left(s\right)\cdot {\tilde{n}}_{I}\left(t\right)\right\} & =E\left\{{\tilde{n}}_{I}\left(t+\tau \right)\cdot {\tilde{n}}_{I}\left(t\right)\right\}=\phi_{I}^{noise}\left(\tau \right)\\ E\left\{{\tilde{n}}_{I}\left(s\right)\cdot {\tilde{n}}_{Q}\left(t\right)\right\} & =E\left\{{\tilde{n}}_{I}\left(s\right)\right\}\cdot E\left\{{\tilde{n}}_{Q}\left(t\right)\right\}=0\cdot 0=0\\ E\left\{{\tilde{n}}_{Q}\left(s\right)\cdot {\tilde{n}}_{I}\left(t\right)\right\} & =E\left\{{\tilde{n}}_{Q}\left(s\right)\right\}\cdot E\left\{{\tilde{n}}_{I}\left(t\right)\right\}=0\cdot 0=0\\ E\left\{{\tilde{n}}_{Q}\left(s\right)\cdot {\tilde{n}}_{Q}\left(t\right)\right\} & =E\left\{{\tilde{n}}_{Q}\left(t+\tau \right)\cdot {\tilde{n}}_{Q}\left(t\right)\right\}=\phi_{Q}^{noise}\left(\tau \right). \end{array} \end{array}$$

Note that the fact that noise is stationary and that its in-phase and quadrature components are independent (Section 4) has been taken into account. On the other hand, from [23] we have $\phi_{I}^{noise}\left(\tau \right)=\phi_{Q}^{noise}\left(\tau \right)=\phi_{\tilde{n}}\left(\tau \right)$. Then, based on these preliminary results, the expected value of $I_{2}\cdot I_{5}$ can be initially written as follows:

(A12) $$\begin{array}{cc} E\left\{I_{2}\cdot I_{5}\right\} & =4E\{\left({\tilde{x}}_{I}\left(s\right)\cdot h_{re}\left(s\right)-{\tilde{x}}_{Q}\left(s\right)\cdot h_{im}\left(s\right)\right)\\ \; & \cdot \left({\tilde{x}}_{I}\left(t\right)\cdot h_{re}\left(t\right)-{\tilde{x}}_{Q}\left(t\right)\cdot h_{im}\left(t\right)\right)\}\cdot \phi_{\tilde{n}}\left(\tau \right)\\ \; & +4E\{\left({\tilde{x}}_{I}\left(s\right)\cdot h_{im}\left(s\right)+{\tilde{x}}_{Q}\left(s\right)\cdot h_{re}\left(s\right)\right)\\ \; & \cdot \left({\tilde{x}}_{I}\left(t\right)\cdot h_{im}\left(t\right)+{\tilde{x}}_{Q}\left(t\right)\cdot h_{re}\left(t\right)\right)\}\cdot \phi_{\tilde{n}}\left(\tau \right). \end{array}$$

Next, by performing standard calculations, the latter equation can be rewritten in this way:

(A13) $$\begin{array}{cc} E\left\{I_{2}\cdot I_{5}\right\} & =4E\left\{ℜ\left\{\tilde{x}\left(s\right)\cdot h\left(s\right)\right\}ℜ\left\{\tilde{x}\left(t\right)\cdot h\left(t\right)\right\}\right\}\cdot \phi_{\tilde{n}}\left(\tau \right)\\ \; & +4E\left\{ℑ\left\{\tilde{x}\left(s\right)\cdot h\left(s\right)\right\}ℑ\left\{\tilde{x}\left(t\right)\cdot h\left(t\right)\right\}\right\}\cdot \phi_{\tilde{n}}\left(\tau \right). \end{array}$$

Here, *ℑ* stands for the imaginary part. Given the linearity of the expectation operator, the latter result can be further simplified:

(A14) $$\begin{array}{cc} E\left\{I_{2}\cdot I_{5}\right\} & =4\phi_{\tilde{n}}\left(\tau \right)\\ \; & \cdot E\{ℜ\left\{\tilde{x}\left(s\right)\cdot h\left(s\right)\right\}ℜ\left\{\tilde{x}\left(t\right)\cdot h\left(t\right)\right\}\\ \; & +ℑ\left\{\tilde{x}\left(s\right)\cdot h\left(s\right)\right\}ℑ\left\{\tilde{x}\left(t\right)\cdot h\left(t\right)\right\}\}\\ \; & =4\phi_{\tilde{n}}\left(\tau \right)E\left\{ℜ\left\{\tilde{x}\left(s\right)\cdot h\left(s\right){\left(\tilde{x}\left(t\right)\cdot h\left(t\right)\right)}^{*}\right\}\right\}. \end{array}$$

Recall that the asterisk denotes the complex conjugate operator. Now, we can replace *s* by t+τ and formulate $E\left\{I_{2}\cdot I_{5}\right\}$ in terms of correlations

(A15) $$\begin{array}{cc} E\left\{I_{2}\cdot I_{5}\right\} & =4\phi_{\tilde{n}}\left(\tau \right)\\ \; & \cdot E\left\{ℜ\left\{\tilde{x}\left(t+\tau \right)\cdot h\left(t+\tau \right){\left(\tilde{x}\left(t\right)\cdot h\left(t\right)\right)}^{*}\right\}\right\}\\ \; & =4\phi_{\tilde{n}}\left(\tau \right)ℜ\left\{E\left\{\tilde{x}\left(t+\tau \right)\cdot {\tilde{x}}^{*}\left(t\right)\cdot h\left(t+\tau \right)\cdot h{\left(t\right)}^{*}\right\}\right\}\\ \; & =4\phi_{\tilde{n}}\left(\tau \right)ℜ\left\{E\left\{\tilde{x}\left(t+\tau \right)\cdot {\tilde{x}}^{*}\left(t\right)\right\}\cdot E\left\{h\left(t+\tau \right)\cdot h{\left(t\right)}^{*}\right\}\right\}\\ \; & =4\phi_{\tilde{n}}\left(\tau \right)ℜ\left\{2\phi_{\tilde{x}}\left(t,\tau \right)2\phi_{h}\left(\tau \right)\right\}\\ \; & =16\phi_{\tilde{n}}\left(\tau \right)ℜ\left\{\phi_{\tilde{x}}\left(t,\tau \right)\phi_{h}\left(\tau \right)\right\}. \end{array}$$

Note from [25] that, in the complex field, $\phi_{U\cdot V}=\frac{1}{2}E\left\{U\cdot V^{*}\right\}$.

Product $I_{2}\cdot I_{6}$

(A16)
$$\begin{array}{cc} I_{2}\cdot I_{6} & =2\left({\tilde{n}}_{I}^{2}\left(t\right)+{\tilde{n}}_{Q}^{2}\left(t\right)\right)\\ \; & \cdot (\left({\tilde{x}}_{I}\left(s\right)\cdot h_{re}\left(s\right)-{\tilde{x}}_{Q}\left(s\right)\cdot h_{im}\left(s\right)\right)\cdot {\tilde{n}}_{I}\left(s\right)\\ \; & +\left({\tilde{x}}_{I}\left(s\right)\cdot h_{im}\left(s\right)+{\tilde{x}}_{Q}\left(s\right)\cdot h_{re}\left(s\right)\right)\cdot {\tilde{n}}_{Q}\left(s\right)). \end{array}$$

Now, we can determine the corresponding expected value by making use of the independence properties already outlined:

(A17) $$\begin{array}{cc} E\left\{I_{2}\cdot I_{6}\right\} & =2E\left\{\left({\tilde{x}}_{I}\left(s\right)\cdot h_{re}\left(s\right)-{\tilde{x}}_{Q}\left(s\right)\cdot h_{im}\left(s\right)\right)\right\}\\ \; & \cdot E\left\{\left({\tilde{n}}_{I}^{2}\left(t\right)+{\tilde{n}}_{Q}^{2}\left(t\right)\right)\cdot {\tilde{n}}_{I}\left(s\right)\right\}\\ \; & +2E\left\{\left({\tilde{x}}_{I}\left(s\right)\cdot h_{im}\left(s\right)+{\tilde{x}}_{Q}\left(s\right)\cdot h_{re}\left(s\right)\right)\right\}\\ \; & \cdot E\left\{\left({\tilde{n}}_{I}^{2}\left(t\right)+{\tilde{n}}_{Q}^{2}\left(t\right)\right)\cdot {\tilde{n}}_{Q}\left(s\right)\right\}. \end{array}$$

Note that this formula relies on four expectations that only involve noise, namely $E\left\{{\tilde{n}}_{I}^{2}\left(t\right)\cdot {\tilde{n}}_{I}\left(s\right)\right\}$, $E\left\{{\tilde{n}}_{Q}^{2}\left(t\right)\cdot {\tilde{n}}_{I}\left(s\right)\right\}$, $E\left\{{\tilde{n}}_{I}^{2}\left(t\right)\cdot {\tilde{n}}_{Q}\left(s\right)\right\}$ and $E\left\{{\tilde{n}}_{Q}^{2}\left(t\right)\cdot {\tilde{n}}_{Q}\left(s\right)\right\}$. Two of them are zero as a result of the statistical independence between the in-phase and quadrature components of the equivalent low-pass noise signal:

(A18) $$E\left\{{\tilde{n}}_{Q}^{2}\left(t\right)\cdot {\tilde{n}}_{I}\left(s\right)\right\}=E\left\{{\tilde{n}}_{Q}^{2}\left(t\right)\right\}\cdot E\left\{{\tilde{n}}_{I}\left(s\right)\right\}=N_{R}\cdot 0=0;$$

(A19) $$E\left\{{\tilde{n}}_{I}^{2}\left(t\right)\cdot {\tilde{n}}_{Q}\left(s\right)\right\}=E\left\{{\tilde{n}}_{I}^{2}\left(t\right)\right\}\cdot E\left\{{\tilde{n}}_{Q}\left(s\right)\right\}=N_{R}\cdot 0=0.$$

On the other hand, because ${\tilde{n}}_{I}\left(t\right)$ is a zero-mean normal random process, any set of random variables obtained from it by selecting particular time instants constitute a zero-mean multivariate normal random vector. Hence, it satisfies the Isserlis’ theorem [26], which states that any odd-order moment of a zero-mean multivariate normal random vector is zero. Accordingly,

(A20) $$E\left\{{\tilde{n}}_{I}^{2}\left(t\right)\cdot {\tilde{n}}_{I}\left(s\right)\right\}=0.$$

The same statements can be formulated about process ${\tilde{n}}_{Q}\left(t\right)$ to conclude that

(A21) $$E\left\{{\tilde{n}}_{Q}^{2}\left(t\right)\cdot {\tilde{n}}_{Q}\left(s\right)\right\}=0.$$

Thus, we definitely have:

(A22) $$E\left\{I_{2}\cdot I_{6}\right\}=0.$$

Product $I_{3}\cdot I_{4}$

As this product is formally equivalent to $I_{1}\cdot I_{6}$, we have

(A23) $$E\left\{I_{3}\cdot I_{4}\right\}=E\left\{I_{1}\cdot I_{6}\right\}.$$

Product $I_{3}\cdot I_{5}$

This product is formally equivalent to $I_{2}\cdot I_{6}$. Thus,

(A24) $$E\left\{I_{3}\cdot I_{5}\right\}=0.$$

Product $I_{3}\cdot I_{6}$

(A25)
$$I_{3}\cdot I_{6}=\left({\tilde{n}}_{I}^{2}\left(s\right)+{\tilde{n}}_{Q}^{2}\left(s\right)\right)\cdot \left({\tilde{n}}_{I}^{2}\left(t\right)+{\tilde{n}}_{Q}^{2}\left(t\right)\right).$$

Its expectation is as follows:

(A26) $$\begin{array}{cc} E\left\{I_{3}\cdot I_{6}\right\} & =E\left\{\left({\tilde{n}}_{I}^{2}\left(s\right)+{\tilde{n}}_{Q}^{2}\left(s\right)\right)\cdot \left({\tilde{n}}_{I}^{2}\left(t\right)+{\tilde{n}}_{Q}^{2}\left(t\right)\right)\right\}\\ \; & =E\left\{{\tilde{n}}_{I}^{2}\left(s\right)\cdot {\tilde{n}}_{I}^{2}\left(t\right)\right\}+E\left\{{\tilde{n}}_{I}^{2}\left(s\right)\cdot {\tilde{n}}_{Q}^{2}\left(t\right)\right\}\\ \; & +E\left\{{\tilde{n}}_{Q}^{2}\left(s\right)\cdot {\tilde{n}}_{I}^{2}\left(t\right)\right\}+E\left\{{\tilde{n}}_{Q}^{2}\left(s\right)\cdot {\tilde{n}}_{Q}^{2}\left(t\right)\right\}. \end{array}$$

This expression contains two types of expectations—those that involve both the in-phase and quadrature components of noise, and those that only involve one of these components. Regarding the latter, we can substitute *s* by t+τ and formulate them as auto-correlations, whereas for the former we can make use of the properties highlighted in Section 4:

(A27) $$\begin{array}{cc} E\left\{I_{3}\cdot I_{6}\right\} & =E\left\{{\tilde{n}}_{I}^{2}\left(t+\tau \right)\cdot {\tilde{n}}_{I}^{2}\left(t\right)\right\}\\ \; & +E\left\{{\tilde{n}}_{I}^{2}\left(s\right)\right\}\cdot E\left\{{\tilde{n}}_{Q}^{2}\left(t\right)\right\}\\ \; & +E\left\{{\tilde{n}}_{Q}^{2}\left(s\right)\right\}\cdot E\left\{{\tilde{n}}_{I}^{2}\left(t\right)\right\}\\ \; & +E\left\{{\tilde{n}}_{Q}^{2}\left(t+\tau \right)\cdot {\tilde{n}}_{Q}^{2}\left(t\right)\right\}\\ \; & =\phi_{{\tilde{n}}_{I}^{2}}\left(\tau \right)+N_{R}\cdot N_{R}+N_{R}\cdot N_{R}+\phi_{{\tilde{n}}_{Q}^{2}}\left(\tau \right)\\ \; & =2N_{R}^{2}+\phi_{{\tilde{n}}_{I}^{2}}\left(\tau \right)+\phi_{{\tilde{n}}_{Q}^{2}}\left(\tau \right). \end{array}$$

Next, by making use of the Isserlis’ theorem for the even-order moments [26], we have

(A28) $$\begin{array}{cc} \phi_{{\tilde{n}}_{I}^{2}}\left(\tau \right) & =E\left\{{\tilde{n}}_{I}^{2}\left(t+\tau \right)\cdot {\tilde{n}}_{I}^{2}\left(t\right)\right\}\\ \; & =E\left\{{\tilde{n}}_{I}\left(t+\tau \right)\cdot {\tilde{n}}_{I}\left(t+\tau \right)\cdot {\tilde{n}}_{I}\left(t\right)\cdot {\tilde{n}}_{I}\left(t\right)\right\}\\ \; & =E\left\{{\tilde{n}}_{I}\left(t+\tau \right)\cdot {\tilde{n}}_{I}\left(t+\tau \right)\right\}\cdot E\left\{{\tilde{n}}_{I}\left(t\right)\cdot {\tilde{n}}_{I}\left(t\right)\right\}\\ \; & +E\left\{{\tilde{n}}_{I}\left(t+\tau \right)\cdot {\tilde{n}}_{I}\left(t\right)\right\}\cdot E\left\{{\tilde{n}}_{I}\left(t+\tau \right)\cdot {\tilde{n}}_{I}\left(t\right)\right\}\\ \; & +E\left\{{\tilde{n}}_{I}\left(t+\tau \right)\cdot {\tilde{n}}_{I}\left(t\right)\right\}\cdot E\left\{{\tilde{n}}_{I}\left(t+\tau \right)\cdot {\tilde{n}}_{I}\left(t\right)\right\}\\ \; & =N_{R}\cdot N_{R}+2{\left(\phi_{I}^{noise}\left(\tau \right)\right)}^{2}=N_{R}^{2}+2\phi_{\tilde{n}}^{2}\left(\tau \right). \end{array}$$

Similarly:

(A29) $$\begin{array}{cc} \phi_{{\tilde{n}}_{Q}^{2}}\left(\tau \right) & =N_{R}^{2}+2\phi_{\tilde{n}}^{2}\left(\tau \right). \end{array}$$

Next, by introducing Equations (A28) and (A29) into Equation (A27), and making some additional manipulations, we can obtain the following result:

(A30) $$E\left\{I_{3}\cdot I_{6}\right\}=4N_{R}^{2}+4\phi_{\tilde{n}}^{2}\left(\tau \right).$$

Finally, from Equations (A4), (A6), (A8), (A9), (A15), (A22), (A23), (A24) and (A30), we can derive the following expression for $E\left\{∥\tilde{r}{\left(s\right)∥}^{2}{∥\tilde{r}\left(t\right)∥}^{2}\right\}$, with s=t+τ:

(A31) $$\begin{array}{cc} E\left\{∥\tilde{r}{\left(t+\tau \right)∥}^{2}{∥\tilde{r}\left(t\right)∥}^{2}\right\} & =\phi_{∥\tilde{x}{∥}^{2}}\left(t,\tau \right)\phi_{{∥h∥}^{2}}\left(\tau \right)\\ \; & +4N_{R}\cdot E\left\{∥\tilde{x}{\left(t\right)∥}^{2}\right\}E\left\{{∥h\left(t\right)∥}^{2}\right\}\\ \; & +16\phi_{\tilde{n}}\left(\tau \right)\cdot ℜ\left\{\phi_{\tilde{x}}\left(t,\tau \right)\phi_{h}\left(\tau \right)\right\}\\ \; & +4N_{R}^{2}+4\phi_{\tilde{n}}^{2}\left(\tau \right). \end{array}$$
